# Shared medical appointments in English primary care for long-term conditions: a qualitative study of the views and experiences of patients, primary care staff and other stakeholders

**DOI:** 10.1186/s12875-022-01790-z

**Published:** 2022-07-20

**Authors:** Fiona Graham, Helen Martin, Jan Lecouturier, Amy O’Donnell, Mei Yee Tang, Katherine Jackson, Falko F. Sniehotta, Eileen Kaner

**Affiliations:** 1 Policy Research Unit Behavioural Science, Population Health Sciences Institute, Faculty of Medical Sciences, Newcastle University, Baddiley Clark Building, Richardson Road, NE1 4AX Newcastle upon Tyne, UK; 2 Research & Evidence Team, NECS, Riverside House, Goldcrest Way, NE15 8NY Newcastle upon Tyne, UK; 3 University of Twente, Faculty of Behavioural, Management and Social Sciences Cubicus, P.O. Box 217, AE 7500 Enschede, Germany; 4 Department of Public Health, Social and Preventative Medicine, Heidelberg University, Tridomus B, Level 5, Ludolf-Krehl-Strasse, 7-11, 68167 Mannheim, Germany

**Keywords:** Shared medical appointments, Group consultations, Primary care, General practice, Implementation, Semi-structured interviews

## Abstract

**Background:**

Shared medical appointments (SMAs) or group consultations have been promoted in primary care to improve workload pressures, resource-use efficiency and patient self-management of long-term conditions (LTCs). However, few studies have explored stakeholders’ perspectives of this novel care delivery model in the English NHS context, particularly patients’ views and experiences of SMAs.

**Method:**

Semi-structured interviews were used to explore the perspectives of stakeholders (21 patients, 17 primary care staff, 2 commissioners and 2 SMA training providers) with and without SMA experience from a range of geographical and socio-economic backgrounds in the North East and North Cumbrian region of England. Thematic analysis was conducted to examine perceptions around impact on patient care and outcomes and barriers and facilitators to implementation.

**Results:**

Three main themes were identified: ‘Value of sharing’, ‘Appropriateness of group setting’, ‘Implementation processes’. Patients experiences and perceptions of SMAs were largely positive yet several reported reservations about sharing personal information, particularly in close-knit communities where the risk of breaching confidentiality was perceived to be greater. SMAs were considered by patients and staff to be inappropriate for certain personal conditions or for some patient groups. Staff reported difficulties engaging sufficient numbers of patients to make them viable and having the resources to plan and set them up in practice. Whilst patients and staff anticipated that SMAs could deliver high quality care more efficiently than 1:1 appointments, none of the practices had evaluated the impact SMAs had on patient health outcomes or staff time.

**Conclusion:**

Stakeholder experiences of SMA use in English primary care are largely similar to those reported in other countries. However, several important cultural barriers were identified in this setting. Further work is needed to better understand how patient and staff perceptions, experiences and engagement with SMAs change with regular use over time. Concerns regarding staff capacity, additional resource requirements and numbers of eligible patients per practice suggest SMAs may only be feasible in some smaller practices if facilitated by primary care networks. Further mixed-method evaluations of SMAs are needed to inform the evidence base regarding the effectiveness, efficiency and feasibility of SMAs long-term and subsequently their wider roll-out in English primary care.

**Supplementary Information:**

The online version contains supplementary material available at 10.1186/s12875-022-01790-z.

## Introduction

Innovative care delivery models to support patient self-management of long-term conditions (LTCs) are at the centre of government healthcare policies worldwide, including National Health Service in England and Wales [1]. One model of care proposed to improve resource use efficiency in primary care and patient self-management are group consultations, also known as shared medical appointments (SMAs) [2, 3]. A recent systematic review of SMAs in primary care found they are at least as effective as usual care with regards to health outcomes and showed potential in improving self-efficacy which may enhance self-care [4]. To support the adoption and implementation of this model, it is important to understand whether patients and providers consider them acceptable and feasible in practice.

SMAs are an alternative to 1:1 appointments whereby groups of patients with the same LTC(s) share an appointment and receive clinical management, patient education and peer support. SMAs can be delivered face-to-face or online (video SMAs) and are usually co-delivered by a clinician(s) (usually GP, Nurse Practitioner or Pharmacist) alongside a facilitator (non-clinical member of practice staff) and typically last up to 90 min. Practice staff have reported SMAs offer advantages over 1:1 appointment for the patients including peer support and accountability, as well as practice benefits in terms of efficiency and the longer appointment time enabling the opportunity to provide comprehensive patient-led [5]. Patients have reported some benefits include vicarious learning, though some patients report that they dislike group setting, and report access issues due to the longer appointment time. However most evidence to date is from North America and focuses on provider perspectives and the views of low-income patient groups that may have less relevance to other health systems or patient population [5, 6].

In England and Wales, there have been local initiatives encouraging the use of SMAs in primary care for LTC management that have primarily taken the form of training for practice staff. Training programmes provide primary care teams with a standardised SMAs model format that can be adapted according to local needs and resources. Whilst it has been reported that over 100 practices across England have attended SMA training [3], the extent to which practices have successfully used the model is unclear. A review of the wider literature identified several barriers to implementation including administrative and resource challenges, patient resistance and suitability and role adjustments and uncertainties [7]. Factors identified as supporting successful implementation in primary care include having leadership, teamwork and communication, alongside staff skilled in group facilitation [7]. The experiences of adopting and delivering SMAs in English primary care requires further investigation to understand if they are acceptable and feasible to patients and practitioners, and if so, how best to optimise the adoption and implementation of SMAs in primary care. Exploring the views of those involved in commissioning and delivering the training will help to better understand the readiness of the NHS to adopt and embed SMAs in primary care and what resource and support is needed if deemed acceptable and feasible.

## Methods

### Aim

This study aimed to answer the following questions:

- What are the views and experiences of patients, practice staff, commissioners and SMA training programme providers about using SMAs in primary care for patients with LTC?
- What are the perceived barriers and facilitators to implementation and maintenance?

### Design

This was a qualitative study using semi-structured interviews.

### Setting and participants

The study was conducted in the North East and North Cumbria region of England that covers a wide range of geographical settings from major urban to rural, with a lower percentage of ethnic minorities than the English average, and higher levels of deprivation in 13 out of 18 local authority areas [8]. In 2018, Health Education England commissioned in-person training for primary care teams. The training was primarily for practice staff who will be organising (practice administrators and managers) and delivering SMAs (nurse practitioners and GPs) and SMA facilitators (non-clinical practice staff- usually administrators/ managers). When this study commenced, staff from a total of 67 (19%) general practices in this region had attended SMA training.

To obtain a broad range of perspectives about SMAs, a purposive sampling strategy was used based upon SMA experience. This included patients with LTCs (registered with practices who had attended SMA training) who had either accepted or declined an invitation to attend an SMA; patients with LTCs with no SMA experience that could be invited to attend an SMA in future; primary care staff (practice-based and commissioners) who had no prior awareness of SMAs; staff who had attended SMA training in 2018; staff who had run SMAs in general practice for LTCs and senior members of the SMA learning support programme (trainers).

### Identification and recruitment

North of England Care Support (NECS) identified practices whose staff had attended SMA training in 2018. Practices were purposively selected based on their geographical location and area deprivation level to ensure the sample included a broad range of perspectives including participants living and working in urban and rural locations in both socio-economically deprived and affluent areas. Managers of these practices were approached with information about the study by email, followed up by telephone calls and face-to-face meetings to request their support with recruitment of practice staff and patients to the study. Managers were asked to identify and forward study information to practice staff involved in the delivery of SMAs or who could be involved in their delivery in future. Staff interested in participating in the study contacted the research team to schedule the interview. Commissioners involved in supporting the use of SMAs in the North of England were identified through local networks.

Participating practice staff identified eligible patients through a search of their records of patients invited to attend an SMA and from personal recollection. Study invitation packs were posted or handed to participants attending the SMA. Patients willing to participate in the study contacted the research team. Patients were then screened for eligibility and interviews were arranged. To recruit patients with LTCs without SMA experience, PPI representatives affiliated with the Policy Research Unit in Behavioural Science at Newcastle University were asked to share the study invitation with people in their networks and obtain their consent to be contacted by the research team. SMA trainers were identified from their website and invited by email to participate in an interview.

### Data collection

Interview guides were developed to explore stakeholder views of SMAs and their impact on patient care and patient outcomes, experiences of delivering this mode of care, and barriers and facilitators to attendance/delivery (Additional file 1: Appendix). Interviews were conducted in-person, by telephone or via the video conferencing software Zoom as per patient preference. Interviews ranged in length from approx. 20–75 min. Recruitment stopped when the study team felt data adequacy was reached i.e. there was enough data and commonalities across codes to meet the aims of the research [9].

### Researcher characteristics and reflexivity

Interviews were conducted by two researchers (FG interviewed patients, NHS staff and SMA trainers, HM interviewed NHS staff) who were of the same nationality and ethnicity as most participants (White British). None of the authors have participated in an SMA as a patient or practitioner, nor have they observed an SMA taking place. However, several have conducted research in this field for 1–3 years thus are familiar with the literature and listening to the accounts of patients and health professionals which may have influenced their interpretation of the findings.

### Analysis

All interviews were audio recorded, transcribed verbatim and then anonymised. The data were analysed thematically following the approach outlined by Braun and Clarke [10]. In brief, this included reading and re-reading the transcripts to familiarise oneself with the data. From reading the transcripts, data pertinent to the research questions were coded inductively line by line. These codes were grouped into themes and then into subthemes where appropriate. *Themes were reviewed and defined and eventually named.* All the transcripts were first coded by one researcher (FG) and 20% were independently double-coded (JL/HM). The wider research team also met to discuss and agree the final themes, sub-themes and definition of each theme. Nvivo version 12 [11] was used to support the organisation of the data during the coding process.

## Results

A total of 39 interviews were conducted either in-person (*n* = 19), via Zoom (*n* = 11) or by telephone (*n* = 9), between October 2019- October 2020.

The sample included 21 patients with LTCs, 13 women, 7 men, 1 transgender person, with an age range between 38 – 87 years (See Table 1). Patients were from a range of socio-economic backgrounds, residing in areas with the lowest and highest levels of multiple deprivation (indices of multiple deprivation decile 10 and 1, respectively). Most patient participants were retired, White British and had experience of SMAs. The condition for which most patients attended an SMA was high cholesterol with risk of familial hypercholesterolemia. Patients with SMA experience had attended a single, one-off SMA, were mostly female, and retired. Of the seven participants without SMA experience, five had not been invited to attend an SMA, and two that had been invited but were unable to as the time was unsuitable.

**Table 1:** SMA participant characteristics (patients)

**Patient participants (*n* = 21)**	
**Age (yrs)**	
Range	38–87
Mean (SD)	64
**Gender**	
Men	7
Women	13
Transgender	1
**Index of Multiple Deprivation (***n***)**	
Decile 1–5 (Highest deprivation)	10
Decile 6–10 (Lowest deprivation)	10
Missing	1
**Ethnicity**	
White	20
Mixed	1
**Marital Status**	
Married	14
Widowed	3
Single	4
**Occupation**	
Retired	12 (57%)
Full-time employed	2 (10%)
Part-time employed	3 (14%)
Self-employed	2 (10%)
Long term sick	1 (5%)
Carer	1 (5%)
**Highest educational qualification**	
Level 2 (GCE, GCSE, O-levels)	4
Level 3 (AS level, A-Levels	4
Level 4 (CertHE, Higher National Certificate)	3
Level 5 (Diploma of higher education, High national diploma)	0
Level 6 (degree apprenticeship, degree with honours)	5
Level 7 (Master’s degree, post-graduate certificate)	1
Level 8 (PhD or DPhil)	3
**Number of chronic conditions**	
0 (at risk)	4
1	4
2	3
3 or more	10
**Chronic conditions**	
High Cholesterol/ Risk of Familial Hypercholesterolaemia (FH)	10
Diabetes /prediabetes	9
Hypertension	4
Asthma	2
Depression	2
Chronic pain/ Fibromyalgia	2
Hyper/Hypothyroidism	2
Osteoporosis	1
Arthritis	1
Parkinson’s Disease	1
Chronic Fatigue Syndrome	1
**SMA experience (attended SMA)**	14
Women	10
Men	4
Retired	9
Full-time employed	3
Part-time employed	1
Carer	1
**No SMA experience**	7
Not invited	5
Invited -unable to attend	2

A total of sixteen NHS staff were recruited, thirteen had SMA experience: seven had attended SMA training and subsequently delivered SMAs; two had attended training but were not involved in SMA delivery; and two had delivered SMAs but had not attended any formal training. Seven staff had no SMA delivery experience, this included: two GPs; two practice manager; two commissioners; and one social prescriber. See Table 2 for provider characteristics; note that the details of the training providers have been omitted from the table to retain anonymity.

**Table 2:** Provider characteristics

**NHS staff (*n*** **= 16)**	
**Gender**	
Men	5
Women	11
**Ethnicity**	
White	16
**NHS staff role**	
GP	5
Practice Manager	3
Administrator	2
Nurse Practitioner	2
Commissioner	2
Pharmacist	1
Social prescriber	1
**SMA experience (*n*** **= 13)**	
**Attended training and delivered SMA**	
Practice manager	2
Nurse practitioner	2
Pharmacist	1
Administrator	1
GP	1
**Attended training (no delivery experience)**	
Practice manager	1
Commissioner	2
Social prescriber	1
**Delivered SMA (no formal training)**	
GP (1 female, 1 male)	2
**No SMA experience (*n*= 3)**	
Did not attend training:	
GP (2 male)	2
Practice manager	1

Practice-based NHS staff in the sample worked in eight different general practices in the NENC, characteristics of these practices and their SMAs use are outlined in Table 3. Most practices had run at least one SMA but had since stopped. Only two of the practices that took part in this study had successfully set-up and were regularly running SMAs to deliver routine care. These practices were replacing 1:1 annual review appointments for patients with LTCs including asthma, high cholesterol, diabetes and COPD with SMAs.

**Table 3:** Characteristics of SMA and their use in practices

**Practice**	**SMA condition(s)**	**No. SMAs delivered**	**SMA status at interview**	**Staff delivering SMA**		**Practice list size (patients)**	**Setting (deprivation level**^**b**^**)**
				**Clinician**	**Facilitator**		
**A**	risk of familial hypercholesterolemia	7	not running	GP	manager/ administrator	7,000	rural (HD)
**B**	diabetes, asthma, high cholesterol	> 25	using regularly	Nurse/external clinician	manager/ administrator	10,000	major urban (LD)
**C**	diabetes	1	not running	nurse	administrator	13,000	major urban (HD)
**D**	multi-morbidities	6	using regularly	nurse	healthcare assistant	16,000	major urban (HD)
**E**	high cholesterol, asthma, COPD	> 25	using regularly	pharmacist/ GP/nurses	unclear	20,000	major urban (HD)
**F**	diabetes	1	just started	nurse	healthcare assistant	36,000	city & town (HD)
**G**^a^	chronic pain	> 25	yes – trialling video SMAs)	GP/ pharmacist/ nurse	unclear	12,000	major urban (HD)
**H**^a^	multimorbidity to include: overweight / obesity / hypertension, pre-diabetes / type 2 diabetes	6	not running	GP	healthcare assistant	12,000	city & town (HD)

*GP* general practitioner

^a^Interviewed during height of pandemic

^b^*HD* Higher deprivation (Indices of multiple deprivation deciles 1–5), *LD *Lower deprivation (Indices of multiple deprivation deciles 6–10)

Three overarching themes were identified that captured stakeholder views and experiences of delivering/attending SMAs for LTCs. These were: ‘Value of sharing; ‘Appropriateness of group setting’; and ‘Implementation processes'. These themes and associated sub-themes are illustrated in Table 4. The following abbreviations are used from here: S = Staff (includes practice-based staff and commissioners), P = Patients and T = Training providers, MM = Multimorbidity, SE = SMA experience, NSE = No SMA experience, HD = High deprivation, LD = Low deprivation.

**Table 4:** Themes and subthemes

Theme	Subthemes
Value of sharing	Overcomes feeling of isolation and supports self-care Time for learning Enjoyment of the novelty and greater informality Empowering patients
Appropriateness of group setting	Patient preference and suitability Confidentiality and personal concerns
Implementation processes	Training Capacity and resource Leadership and ‘buy-in’ from colleagues Engagement and attendance Evaluating efficiency

### Value of sharing

This theme captures perceptions and experiences about the benefits that the presence of other patients adds to the appointment in terms of the impact upon the delivery and quality of patient care.

### Overcomes feelings of isolation and supports self-care

All patients and practice staff anticipated that SMAs would provide an opportunity for patients to speak to others with the same condition. This would benefit the patient by helping to validate feelings and experiences of living with an LTC and feel emotionally supported, as described by one patient:

“…I think unless you’ve been there and you’ve experienced chronic pain for however long, you can have all these skills and all the qualifications but you don’t really understand what it’s like to live with a chronic illness day in, day out. So, I think for that aspect, I think people experiencing the same problem, it could be quite supportive.” P20, F, 50-59yrs, NSE, LD

Patients in this sample who had attended an SMA reported experiencing little direct conversation with other patients during or after the session. However, they described that after listening to the conversations between staff and other patients, they felt less isolated having encountered ‘people in the same boat’ (P2, P5, P12). Practice staff reported that patients were able to identify with others with the same condition and therefore engage more with their care. One primary care staff member reflected upon a children’s asthma SMA:

“…they may not have another person within their friendship group who has asthma, but when they come to the group clinic there are other people in their age range who have it. They’re able to identify that and…. get a better understanding of what’s happening and realise they’re not alone.” S6, SE, Urban, HD

GPs without SE expressed reservations about providing individualised results in a group and felt it might require a sensitive discussion with a patient outside the group on a 1–2-1 basis.

“People may or may not understand what different results mean. For example, diabetes. They may just see that a rising HbA1c… is just due to poorly controlled diabetes, but actually it could be due to something else, which is quite a sensitive discussion with a patient and potentially breaking bad news.” S11, NSE, Urban.

However, patients with high cholesterol who had attended an SMA for those at risk of Familial Hypercholesterolemia, reported that despite having some concerns about sharing personal information in the group, they found comparing individual blood cholesterol results with others helpful in understanding their own risk of the condition. A small number also reported that comparing their results with others made them more proactive in the management of their condition.

“it did make me realise…I should make sure I do get my blood test done and checked... If somebody … 20 years older than me, can have [their cholesterol] brought down to a really low level then I could obviously get mine down and make it less harmful to my body.” P10, F, 60-69yrs, SE, LD

At the same time, there were other instances where patients reported that once they realised their risk of FH was low, they disengaged from the discussion and felt the SMA was a waste of time.

“I could have met with the GP for 20 minutes myself… It would have been more tailored to me rather than listening to the illnesses that other people had.” P13, F, 50-59yrs, SE

### Time for learning

Patients without SE anticipated the longer appointment time would allow them more time with the clinician to discuss their condition in greater depth than they could during their usual 1 appointment (1:1 appointments usually last 10–15 min in England). This anticipated benefit was confirmed by those with experience of attending an SMA, especially newly diagnosed patients who described the longer appointment as a “life-line” (P5) because it was less rushed, and they had time to "reflect and think"(P5) about their health and it provided them ‘a chance to focus and feel you could so something about it’ (P11). GPs that did not attend the training but were using, or intended to use SMAs, believed that the SMA model provided them the opportunity to spend longer with their patients enabling a more holistic approach to patient care. GPs also reported that having the opportunity to listen to patients sharing their personal experiences of living with the condition enhanced their understanding of the patient and the challenges they faced.

### Enjoyment of the novelty and greater informality

Most patients and providers who had been involved in SMAs reported that they had enjoyed the experience, and found the session more relaxed than traditional 1:1 consultations. In addition to benefitting patients, practice staff reported that they enjoyed delivering care via SMAs as they were ‘less repetitive’ than one-to-one appointments and liked the variety they added to their daily routine. One manager reported there being a ‘buzz about it in the practice after a SMA session’ (S1, SE, Rural) and another said they felt invigorated (S10, Urban). Practice administrators involved in the delivery of the SMAs (facilitator) reported that they particularly enjoyed being in a different role that enabled them to connect with patients on a more personal level. Training providers reported that part of enjoyment of SMAs comes from the experience of teamwork with colleagues within their practice.

### Empowering patients

An anticipated advantage of the group setting expressed by one patient was that having colleagues and other patients present may make the clinician more attentive during their consultation and less inclined to ‘hurry you on’ P20, F, 50–59 yrs, NSE. Similarly, another patient anticipated that the group setting would give them the power not only to share information about their condition with peers but also with their healthcare professional, and to inform and challenge the professional advice given too.

Clinicians and training providers believed that information shared by other group members was more powerful and helped to engage other participants more than information provided by clinicians.

*“I can talk until I’m blue in the face, and some patients will still not want to have an injection. But when you have got the other patients [saying], “No, it’s brilliant. It really helps you. You must try it.” .... It does change people’s minds.”* T2, SE

### Appropriateness of group setting

This theme encapsulates participant views about the limitations of SMAs use, and their perspectives on which patients and conditions they best suit.

### Patient preference and suitability

Most patients reported that they were happy to participate in a group appointment but some expressed concern that group work might not be appropriate for all patients including those who are shy or have social anxiety. This was exemplified by one participant with autism who reported that they would find the group situation too anxiety provoking:

“I’d feel overwhelmed because of the six to ten people in the room, but also… when it was my turn, everyone’s eyes on me, and watching me… I’d probably forget key things that I need to discuss . . . .” P20, F, 50-59yrs, NSE, LD

Individuals past experiences of group work/ SMAs underpinned their intention to attend an SMA in future. Most attendees said they would attend a future SMA as they believed they would benefit from the social support of others in the group. However, this view was not shared by all. One patient felt that main benefit of the group setting was the potential for social interaction which she did not require:

“. . . if you were struggling with an illness and, say, you were isolated or lonely. There may be benefits to being in the group for that, in those circumstances, but for me, I've got a good group of strong, loyal friends… I don't think I'd benefit.” P13, F, 50-59yrs, SE, HD

Practice staff also reported that the group setting suited some patients more than others. They described instances where patients had asked if they could be seen first in the group so they could leave early, and where a patient had walked out of the session upon realising it was delivered as a group. Whilst others reported they were pleasantly surprised when patients attended that they anticipated would not participate in an SMA. Training providers emphasized that SMAs were not intended to be suitable for all, but felt that if implemented widely in the NHS, they would help to reduce demand for individual GP appointments. Training providers also believed that patients who attended would benefit even if they did not share their experiences with others in the group. However, some practice staff expressed doubts that all patients benefitted, implying that those who were already self-managing their LTC well may not necessarily benefit from hearing from others. As one nurse practitioner reflected upon an SMA for diabetes:

“I think there were three there who were well-controlled ad three who weren’t, and it was good for the people who weren’t controlled, but whether the other people learnt much from it , I don’t know” S5, SE, Urban, LD.

Training providers recommended that all patients should be invited to attend an SMA, though there may be some groups they are not suitable for e.g. people with advanced dementia. They also reported that a ‘common sense approach should be taken’ when it comes to children and families and recommended that groups of patients of similar ages should be seen together. One staff member reported that in her experience, SMAs for asthma did not work well with groups of teenagers as they are adversely influenced by others and less likely to share:

“We did find the teenagers we couldn’t do as a group consultation, because they’re very difficult…[in terms of ] attitudes… You only needed one to throw the group off and so we felt it was better still to be one-on-one with those patients” S6, SE, Urban, HD

At the same time, trainers cautioned against practices inviting only patients they anticipated would ‘get on’ with others. Yet two providers in this study reported that they spent time screening out patients deemed unsuitable, including those they thought would be ‘disruptive’. The implications this has for health inequalities requires further examination.

### Confidentiality and personal concerns

Some patients with SMA experience particularly in rural settings, noted that prior to attending they were concerned that they would recognise other people in the group but were relieved when this was not the case. Whilst attending an SMA for identification of Familial Hypercholesterolemia was considered acceptable by most, future attendance depended upon the condition or personal information that might be divulged during the session.

“The problem is with such a close-knit community, you just have to say your name and people know who you are. If it had been anything a bit more personal, I don't actually think it would work” P13 F, 50-59 yrs, SE, HD

SMA participants reported that there were no confidentiality breaches to their knowledge, but some expressed scepticism about the extent to which the information would remain in the group.

One GP without SE believed that SMAs were inherently problematic in their nature given the high risk of confidentiality breaches by other patients in the group and wanted to know how this would circumvented before proceeding.

“A [confidentiality agreement] form is a form. Unless it comes with a legal follow-up then it’s just someone’s name on a paper. [If] I break confidentiality, I could be in front of the General Medical Council or in front of the courts.” S11,, NSE, Urban

Several staff with SMA experience reported that they were initially concerned that patients would be reluctant to attend group appointments over confidentiality concerns. However, after having run the SMA where patients were asked to sign confidentiality agreements, they did not consider confidentiality to be an issue and reported that patients appeared content to share their test results, family history and medical experiences in front of other patients.

### Implementation processes

This theme relates to the key requirements and challenges predicted or experienced, primarily by providers in setting up and delivering SMAs in English general practice.

### Training

Practice managers believed some staff without SE were reluctant to engage with SMAs as they lacked the confidence to consult in a group, particularly those who had not received training first-hand. SMA trainers and commissioners identified lack of time and capacity of staff to attend and high workload and pressure in primary care as key reasons for low attendance at SMA training:

“It is much easier to get teams of people, in secondary care, together for the training. They can cancel their clinics for an afternoon, and it’s the whole team there…In primary care, there’re just not enough people. There are just not enough GPs, they’re always covering for someone, they’re always under pressure. It’s constant.” T2, SE

With regards to training needs, training providers and practice managers were of the view that the facilitator (non-clinician) required the most training, given that their role was vital to ensuring the SMA ran to time and that the facilitator role was very different to their current job role. However, reflections by clinicians in this sample suggest that they too would benefit from SMA training, as delivering care to a group of patients is a new experience:

“I was somewhat petrified…I'm very much comfortable with that one-on-one, face-to-face scenario. To be put in a roomful of people was very much out of my comfort zone.” S14, SE, Urban

### Capacity and resource

Several challenges to setting-up SMAs in practice were reported by practice staff and echoed by commissioners and training providers. Reports of insufficient time, resource, and space in the practice to hold the group sessions were commonly reported by practice staff. Clinicians and managers reported having a lack of time and "headspace" (S4) after the training to think through the set-up work required, which was the reason they had yet to implement SMAs in their practice. Training providers recognised that SMAs require a significant amount of work to set up but believed that the work required to sustain the SMAs would reduce over time. Alongside initial set-up time, practice staff reported that high staff turnover, specifically the loss of trained staff, were additional key barriers to implementation and sustainability of SMAs. One practice that was regularly running SMAs had a member of staff whose role was dedicated to engaging patients and facilitating the SMAs. However, other practice managers reported they had insufficient funds to create a new role to support SMA coordination which has meant that implementation relied upon the ‘goodwill’ of staff to take on the extra work. Both trainers and staff recognised incentives may be helpful to encourage the use of SMA in practice through the provision of money and resource. Commissioners and staff suggested training opportunities for wider practice staff and additional resource by way of enhanced Primary Care Network (PCN) link workers and Care Coordinators taking on role of SMA facilitators in practices. Commissioners also viewed PCNS as a way of facilitating links with voluntary sectors to provide support with language translation for non-native English-speaking participants.

### Leadership and ‘buy-in’ from colleagues

Practices that have successfully embedded SMAs in routine care reported that having staff in senior leadership positions with a personal interest in SMAs was key to their success. This was echoed by commissioners:

“… what has been key is that there have been one or two people in the practice who are really keen… They've almost kind of stepped forward and nominated themselves as a ….clinical lead in their practice...” S15, NSE, Urban

Convincing colleagues to support the adoption of this new approach was a common implementation barrier reported by practice staff who reported colleagues were resistant to change, did not understand the benefits of SMA or were uncertain about what was expected of them. Training providers also reported that the beliefs of health professionals was often a barrier to implementing SMAs successfully:

“Many clinicians, even to this day, will say, ‘I can’t understand why patients would like to be seen in a group’… Their thinking like that was meaning that …they weren’t being successful because [their mind-set] this was getting in the way for them.” T2 SE

Both training providers and practice staff believed that witnessing an SMA in practice would greatly help with ‘buy-in’ from colleagues. However, trained staff reflected that they had limited capacity, and often no occasion, in which to communicate what they were doing to colleagues in their practice, or other practices.

### Engagement and attendance

Patients reported they attended the SMA as they were curious about whether they had the condition for which the practice had organised the group session (those at risk of hypercholesterolaemia). Several newly diagnosed patients reported that they wanted to learn how to manage their condition, they attended without realising the SMAs were novel. Patients in this sample that did not attend an SMA were full-time employed or carers and did not attend because the SMAs were arranged on a day that was unsuitable for them.

Several staff working in practices with different levels of SMA experience, reported that patients had declined an invitation to participate for various reasons including: expressing preference for 1:1 appointments; that they disliked the change to routine care, disliked group work, that they had insufficient time to attend or it was not convenient. Staff reported they had tried to organise SMA at times they thought would be more suitable for patients to attend, for example after school or work, or during the school holidays, but found this unsuccessful and attendance remained low.

Staff in practices running SMAs recommended advertising widely, providing reassurances to patients, setting expectations, and having enthusiastic staff communicating SMAs effectively. Managers of practices that were running SMAs frequently also reported having familiarity and trust in the clinician helped encourage patients to attend. Training providers reported that uptake is most effective when the practice staff are enthused and informed about how best to invite patients and SMAs are offered as a default appointment option:

“If you go to patients and give them a choice, it’s like, ‘I don’t want to go to a group.’… What we say to healthcare professionals is, “Keep it really simple. It’s the three key messages, you get to spend longer with your clinician, you get connected with people who’ve got similar challenges or conditions as you, and people that attend group consultations do better” T2, SE

Of the practices using SMA regularly, one reported it was difficult to get the same patients to attend a second group appointment. In some instances, patients attended only the preliminary information gathering appointment (e.g. when bloods were taken by a healthcare assistant), and did not attend the subsequent SMA (where results are reviewed by the clinician). They also reported that they struggled to get children patients back for a second asthma SMA session, assuming the patients and families felt there wasn’t a need. Having insufficient numbers of patients with the same condition within the practices (‘critical mass’ T2) was also considered a potential barrier to SMA utility in smaller primary care practices. One health professional reflected:

“I think smaller surgeries of 4,000 patients, for example, they're going to have a smaller group of people with a certain ailment…I think they may struggle to run it on a more regular basis.” S7, SE, Urban

A solution to this issue, suggested by a GP, trainers and commissioners, would be to have practices working together and inviting patients with the same condition from a few different practices within a PCN to attend the same SMA.

### Evaluating efficiency

When exploring stakeholders’ perceptions of the benefits of SMAs, several patients regarded them as a positive way of saving the NHS time and money by enabling the clinician to see several people at once. The potential time and costs saved by SMAs was described as a key reason that practice staff had decided to attend SMA training. Commissioners anticipated that SMAs would help to address some of the practical and resource related challenges they faced in primary care, including limited availability of GPs appointments in relation to the large numbers of patients with LTCs. SMAs were also viewed as a way to avoid the large numbers of repetitive 1:1 appointments (S6) that currently reduces their job satisfaction. However, none of the practice staff in this sample had formally assessed the impact of SMAs on their own time, or on the demand for 1:1 appointments in general, so were unable to confirm time or cost savings. Nevertheless, almost all staff with experience of SMA training believed that with time and experience SMAs would become cost-effective, as more patients attended. As one nurse reported:

“we didn’t get a benefit at all when we [ran an SMA for diabetes] because it didn’t save money for us because we only saw six people. So, I mean, in an hour-and-a-half… It took two people to see them.. where I could’ve just done that myself. If the group had been bigger, then there would’ve been a cost saving for the practice there” S5, SE, Urban, LD

Trainers felt that a large scale, national evaluation of SMAs in primary care was required but were concerned that lack of funding and the additional burden the evaluation work would have on practice staff was a key challenge, though were in the process of developing tools to support practices to evaluate their SMAs.

## Discussion

Most patients in our study viewed or experienced SMAs positively and reported a desire to attend future sessions. This is contrary to primary care staff experiences that reported poor patient engagement with SMAs. Our findings suggest that patient engagement with SMAs depended on a patient’s enjoyment or anxiety about group work based on past experiences and their beliefs about the personal gains, (whether they would benefit from the socialisation and peer support), the condition for which the SMA was being held, (whether it was too private or personal to discuss in a group setting) and how comfortable they felt sharing information with people that they may already know. Most patients with SMA experience in this study had attended only one SMA which may be partly a reflection of the limited and irregular use of SMA by practices in the region. Further work is needed to understand how patient experiences changes with regular SMA use overtime to better understand whether the perceived potential benefits for patients are attained and can be maintained with long-term use.

Most practitioners attributed poor patient engagement to short comings of practice staff communicating SMAs effectively to patients. However, practices that were engaging sufficient numbers of patient to run them regularly reported considerable difficulties in getting the same group of patients to attend subsequent SMAs and were often running them as one-off sessions for different patient groups and conditions. Our findings highlight that for SMAs to be viable, practices need to have sufficient numbers of patients with the same condition and have sufficient resource which may partly explain why the smaller practices were not running SMAs regularly. Other reasons practices were not using them regularly were reportedly due to insufficient staff capacity and resource to dedicate to setting them up, lack of leadership and practice wide buy-in, high turnover of trained staff and insufficient training opportunities. Concerns regarding the legal aspects around confidentiality of SMAs were expressed by GPs without SMA experience highlighting a potential barrier to SMA initiation that requires clarification.

Whilst study participants perceived SMAs to be an effective way of delivering holistic care, none of the practice staff interviewed were able to confirm whether patients’ conditions had actually improved since attending the SMA, and none of the patients reported improvements in their health since attending. Similarly, SMAs were perceived to be a more efficient way of delivering care, yet few practices were using SMAs regularly enough to provide insights about their efficiency and use long-term.

### Strengths, limitations and challenges

This study provides various stakeholder views and experiences of SMAs in North East and North Cumbrian region of England, an area that is under-represented in the wider SMA literature [5]. A key strength of the study is that we have incorporated the views of patients who live in areas with the greatest and lowest levels deprivation in the country and patients with one or more LTCs. In addition, our sample includes the perspectives of staff without SMA experience that are missing from previous studies [5, 7]. This has helped to identify preconceptions about SMAs which may represent potential barriers to system wide implementation. This study would have been strengthened had the views of patients that declined an SMA been captured. However, we were unable to recruit this group to our study. Whilst there is a broad range of perspectives included in the sample, the richness of accounts provided by participants was occasionally limited due to minimal SMA experience partly as a result of infrequent use of SMAs in the region. Including the views of patients and practitioners with experience of having attended/delivered multiple SMAs on a more frequent or regular basis would help determine the feasibility of SMAs use long-term. The majority of patients in this sample are retired, therefore further work is needed to explore whether the longer appointment time is created accessibility problems for those in full-time employment. Data was collected before and during the COVID-19 pandemic during which the delivery of care in primary care changed dramatically and remote consultations became the default, thus perceptions of SMAs may have changed.

### Comparison with existing literature

The positive perceptions and experiences of SMA reported by stakeholders in this study echo those found in the wider literature. Similarly, some of the cultural barriers regarding suitability of SMAs for sharing of personal information in additional to healthcare professional concerns regarding confidentiality breaches and the legal aspects of sharing patient results within the group have also previously been reported as potential drawbacks and limitations of SMAs [5, 12]. Patient concerns about access issues and transportation costs were not spontaneously raised as concerns in this study which may because the majority of those in the study had experience of only attending one SMA which was not considered problematic.

Challenges to implementation of SMAs reported by providers and SMA trainers in this study echo the findings of Swaithes et al. [7], which reported key challenges to the initiation and operationalisation of group consultation in primary care related to the amount of time and resource required to set-up the SMAs in practice. We also found that practice-wide ‘buy-in’ was needed to successfully adopt and support this model, and that staff understanding and attitudes underpinned success. Staff in our study also reported difficulties ‘selling’ SMAs to patients and reported low attendance at subsequent appointments by the same group of patients highlighting uncertainty around the use of SMAs long-term. This echoes the conclusions of Booth et al. [12] that suggests once patients feel that they have obtained information, their motivation for attendance wanes. Whilst most patients in our sample reported they would attend another SMA, further exploration of their use over time is needed.

### Implications for future research and clinical practice

Practice staff and patients raised concerns about the suitability of SMAs for some patients and believed that some patients benefitted from attending SMAs more than others. Practice staff also reported instances where they invited patients based upon who they anticipate would attend an SMA or who would work well in a group. These findings highlight important implications for health inequalities. It is important to consider if any inequalities might arise due to differences in the uptake of SMAs in different patient groups based on ethnicity, rurality, health literacy or access to material resources such as transport or digital technologies. The approach taken by practices to identify and invite patients also requires consideration to ensure this does not worsen health inequalities. Further research is needed to establish which groups of patients should be brought together in SMAs for best effect. Guidance about how to make SMAs more inclusive or tailor them for patient groups is needed, along with consideration of the additional resource this incurs.

Healthcare practitioners were unable to determine whether SMAs reduced demand for 1:1 appointments or saved clinician time. Authors recommend that a standardised evaluation plan is developed for use by practices. This would involve the development of standardised measures of SMA effectiveness and efficiency for LTC, such as patient attendance at SMAs, resource and time requirements, healthcare service use, patient satisfaction and health outcomes. Once agreed, these measures ought to be embedded in routine data collection systems, such as patient medical records to enable natural experience and more agile evaluation.

Practices that were unable to incorporate SMAs into routine practice following the training explained they did not have the resources and capacity to adopt them and that further staff training was needed. As recommended previously [7], implementation planning at the practice level is needed to ensure there is appropriate training, leadership, coordination, and practice-based resource to effectively support SMA delivery. This planning ought to also include quality standard checks to ensure that SMAs are arranged and delivered as planned with consideration of the potential implications for health inequalities. Given the variability in their application within and between practices to date, clarification regarding the content and format of delivery including frequency of SMAs for the same patient groups requires further exploration. Our findings suggest that the incorporation of SMAs as part of routine NHS primary care, may require practices working together in their PCNs to pool resources and patient groups to make them viable. Further work is needed to explore how this innovative approach to care may be operationalised.

## Conclusion

Stakeholder experiences of SMA use in English primary care are largely similar to those reported in other countries. However, we have identified several important cultural barriers in this setting. Most patients with minimal or no SMA experience expressed reservations about sharing personal experiences with known others in the group. Similarly, patients and staff deemed SMA only appropriate for those that enjoyed sharing with others and for common conditions that were not ‘too personal’. Further research is needed to understand how patient and staff experiences and perceptions of SMA change with more regular use overtime. The substantial resource requirements for setting up SMAs in addition to the need for sufficient patient numbers to make them viable suggest that the adoption and implementation of SMAs may only be feasible if facilitated by PCNs. Further mixed-method evaluations of SMAs in English primary care are needed to develop the evidence base regarding SMA effectiveness, efficiency and feasibility and inform wider roll-out.

## Supplementary Information

**Additional file 1.** Interview topic guides for a) patient participants, b) staff

## Data Availability

The datasets generated during the current study are not publicly available as consent to share the data in this way was not obtained from interview participants. Data may be available from the corresponding author on reasonable request.

## Abbreviations

COPD: Chronic obstructive pulmonary disease
GP: General practitioner
NHS: National Health Service
NECS: North of England Care Support
NIHR: National Institute of Health Research
LTC: Long term condition
SMA: Shared medical appointment
UK: United Kingdom
