# Supplementary material for: Shared medical appointments in English primary care for long-term conditions: a qualitative study of the views and experiences of patients, primary care staff and other stakeholders
Source: BMC Prim Care. 2022 Jul 20;23:180. doi: 10.1186/s12875-022-01790-z (PMC9298712; doi:10.1186/s12875-022-01790-z)
Supplement: Supplementary file 1 — Additional file 1. Interview topic guides for a) patient participants, b) staff [file 12875_2022_1790_MOESM1_ESM.docx]

**Appendix 1: Interview topic guides for a) patient participants, b) staff**

**a)**

**Patient interview topic guide**

**Research study: shared medical appointments in primary care**

Introduction

1. Introduce researcher and purpose of the study
2. Obtain consent to proceed and to record the conversation
3. Remind interviewee that all information remains confidential, and that they are free to stop the interview and withdraw at any time.

Researcher to complete demographic form with patient participant

Background

1. Please could you tell me, how long you have been a patient at X Practice?
2. How often do you contact or visit the practice? (probe: contacts in an average month/ year)
3. Who would you typically see at the practice and how long would your appointments last?
4. Please could you briefly tell me about any long term health condition you have, or are you at risk of?
5. Overall, how would you describe the care you receive from X Practice?

All – Views and experiences of shared medical appointments

As you know, in this project we are trying to understand more about patients’ views and experiences of a new way of caring for patients called shared medical appointments, or group consultations as they are sometimes called, my questions from here are mainly focused on this.

1. How did you first hear about shared medical appointments? (who told you about them, when and how?)
2. How were shared medical appointments described to you? What did you think about this? (probe positive and negative)
3. Have you spoken to any family or friends about shared medical appointments? What are their thoughts on them?
4. Please could tell me about your experience of being invited to attend a shared medical appointment?

Probe:

Can you remember how you were invited to the group appointment? Who invited you?

What condition was it for?

When you were invited to the group appointment was it clear what the appointment would involve?

Was this similar to how you have been invited to other appointments? How so / what was different?

Attended/ didn’t attend

Questions for participants who did not attend shared medical appointments

1. Can you tell me a bit about why you decided not to attend the shared medical appointment you were invited to?
2. What concerns do you have about shared medical appointments for your care?
3. What do you think might be good about shared medical appointments for your care?
4. How did you think shared medical appointments may be different from the care your currently receive? (probe positives and negatives of both approaches)
5. What might encourage you to attend shared medical appointments in the future?

For participants who have attended shared medical appointments

1. Can you tell me a bit about why you decided to attend the SMA you were invited to?

Probe: Why did you say yes to the appointment? Did you speak with anyone, partner, friend, family, before deciding? Did you have any particular concerns prior to attending the first one?

1. What did you think might be good about shared medical appointments for your care?
2. What concerns, if any, did you have about shared medical appointments for your care?
3. How did you think SMA may be different from the care your currently receive? (probe positives and negatives of both approaches)
4. Please could you tell me about your experience of attending shared medical appointments?
5. What have been the positive aspects of receiving care in a groups setting?
6. What have been the negative aspects of receiving care in a group setting?
7. How useful have you found the appointments for helping you to manage your own symptoms? (In what ways has it been useful? In what ways has it not been useful?)
8. Would you recommend shared medical appointments to other people in your situation? Would you be happy to be seen on an ongoing basis in shared medical appointments for appointments about your condition / risk factor?

All participants - Final section

1. Do you have any other thoughts or suggestions about shared medical appointments?

End of Interview

Thank respondent for their time and switch off recorder.

Discuss how they feel after the interview, ensure they have a copy of the study patient participant information sheet

**b)**

**Staff interview topic guide**

**Research study: shared medical appointments in primary care**

Introduction

1. Introduce researcher and purpose of the study
2. Obtain consent to proceed and to record the conversation
3. Remind interviewee that all information remains confidential, and that they are free to stop the interview and withdraw at any time.

Background

1. Please could you briefly describe your job role? (probe, key tasks/ areas of specialism, who employed by, years in this job)
2. Please could you tell me about your involvement with group consultations to date? (Probe: attended training to deliver group consultations, organising group consultations, discussing Group consultations with patients and other colleagues, delivering group consultations)

For participants who attended training:

3 Since the training have you delivered group consultations?

If yes, probe: how did this come about, to which patient groups, how were patients invited, how many sessions have been run, how were patients invited and by whom. Who ran the sessions (facilitator and practitioner), How did were the sessions received by patients? (Positive/negative?) How did you feel about delivering the session(s)?

If no, probe: what have been the barriers to running group consultations?

For all participants

4 From your experiences, what do you see as the benefits to patients of group consultations compared to usual care? (probe: are there any specific patient groups you think they will be particularly valuable for?)

5 What do you see as the benefits to the practice of group consultations compared to one to one consultations?

6 What do you see as the benefits to healthcare practitioners of group consultations compared to one to one consultations?

7 What do you think are the disadvantages, if any, for patients of attending group consultations compared to one to one care?

8 What do you see as the challenges for practices of delivering group consultations compared to one to one? (probe: additional resources, staff, extra work/time.)

9 What do you see as the challenges to healthcare practitioners of group consultations compared to one to one consultations?

10 What plans do you/ the practice have for using group consultations in the future? (probe: patients groups, who will deliver them)

11 In your view, what makes group consultations successful? (probe: facilitators delivery, patients contribution, resources)

12 How would you describe the culture regarding group consultations in your practice? Is this something that has been discussed within the practice?

13 Have you spoken directly to patients about attending group consultations? If so, how did this feel? How did the patient react?

14 How do you feel / would you feel working with a group of patients rather than on a 1:1 basis? (if not covered in benefits/ challenges to the individual above)

15 Do you feel you would benefit from any further training, information or support regarding group consultations?

16 Final question - Is there anything else you would like to say about group consultations?

End of Interview

Thank participant for their time and switch off recorder.

Ensure they have a copy of the study Professionals Participant Information Sheet
